# The Interaction of Economic Rewards and Moral Convictions in Predicting Attitudes toward Resource Use

**DOI:** 10.1371/journal.pone.0134863

**Published:** 2015-08-12

**Authors:** Brock Bastian, Airong Zhang, Kieren Moffat

**Affiliations:** 1 The University of New South Wales, School of Psychology, Sydney, Australia; 2 Resources in Society Group, CSIRO Mineral Resources Flagship, Pullenvale, Queensland, Australia; Middlesex University London, UNITED KINGDOM

## Abstract

When people are morally convicted regarding a specific issue, these convictions exert a powerful influence on their attitudes and behavior. In the current research we examined whether there are boundary conditions to the influence of this effect. Specifically, whether in the context of salient economic rewards, moral convictions may become weaker predictors of attitudes regarding resource use. Focusing on the issue of mining we gathered large-scale samples across three different continents (Australia, Chile, and China). We found that moral convictions against mining were related to a reduced acceptance of mining in each country, while perceived economic rewards from mining increased acceptance. These two motivations interacted, however, such that when perceived economic benefit from mining was high, the influence of moral conviction was weaker. The results highlight the importance of understanding the roles of both moral conviction and financial gain in motivating attitudes towards resource use.

## Introduction

Whether it is the logging of old growth forests, deep sea oil drilling or factory farming, these commercial activities not only provide humans with critical resources and promote economic prosperity, they also trigger strong moral objection. Conflicts around the use versus protection of resources are evident across a range of sectors extending from mining to fishing, and even the commercial production of meat (e.g, [1]). Increasingly, these conflicts are understood from a moral perspective [2, 3, 4], with research showing that up to 45% of undergraduate students recognize global warming in ethical terms [5]. It is for this reason that people often form strong moral attitudes (moral convictions) regarding issues surrounding resource use. Such moral convictions tend to reflect a deontological approach to the protection and conservation of natural resources, motivating strong issue resistance, and providing little space for a consideration of personal (and other) economic gain [6, 7, 8]. It is for this reason that these two motivations–moral conviction vs. economic gain–are readily seen as diametrically opposed in driving attitudes toward resource use ([9, 10], although see [11]) and are often cited as underlying conflicts between various stakeholder groups [12, 2]. On one hand, moral convictions are formed around a strong desire to protect natural resources, while on the other, economic incentives motivate a tendency to take account of the resources needs of self and other.

Just as these two motivations may characterize the vested interests of different stakeholder groups, they also both represent fundamental motivations for human thought and action. As such, when economic rewards are salient, even those with strong moral convictions against the exploitation of resources may be motivated to give weight to their own and others economic needs. In the current study, we examined whether the salience of economic rewards may provide a boundary condition for the influence of moral conviction in shaping peoples’ attitudes toward resource use. That is, when economic rewards are salient, moral convictions that interfere with maximizing economic rewards may become less influential.

### Moral conviction

Recent research has highlighted that people sometimes hold strong moral attitudes (moral convictions) regarding specific issues. When they do, these moral convictions lead to greater distancing from, and less cooperation with, those who hold opposing views, as well as a reduced ability to generate solutions to resolve disagreements [13]. Because moral convictions are framed as “oughts” and “shoulds”, they also promote strong intentions to act in line with one’s moral standards. Indeed, individuals who have strong moral convictions are more likely to engage in political activism [14, 15], and are less likely to succumb to the well-known pressures of majority influence and consensus information [16, 17, 18, 19].

Moral convictions are deontological to the extent that they treat specific moral norms or attitudes as absolutes, motivating people to pursue these convictions at the expense of all else, including personal gain. For instance, people who held strong moral convictions about the business practices of Wal-Mart acted in less self-interested ways by paying more for consumer goods [20]. This tendency of focusing on the right or wrong of specific outcomes, compared to weighing the benefits across a broader range of considerations, is also evident in attitudes towards environmental protection. For instance, support for environmental protection is often predicted by the extent to which people value the environment for its own sake (ecocentrism), as opposed to giving weight to the various ways in which it may provide benefit for humans (anthropocentrism; [21]).

### Economic rewards

While moral convictions regarding resource use may motivate an all-or-nothing approach to resource decision-making, even when this comes with significant personal costs, a large body of research suggests that the salience of economic rewards also play an important role. Economic considerations have long been positioned as a primary motivation for human behavior within economic theorizing [22, 23] and within psychology, the prospect of financial gain has been shown to motivate a wide range of attitudes and behavior (e.g., [24]). Economic motivations have also been directly tied to environmental attitudes. Focusing on a large cross-national sample, Franzen and Meyer (2010 [25]) found that national wealth was positively related to environmental concern, suggesting that where economic needs are greatest, people are less likely to extend concern to environmental issues (see also [26]; although for a different perspective see [27, 28]).

Economic motivations may also override the inclination to act in accordance with one’s moral standards. For example, it is now well established that people may act in ways that are inconsistent with their moral principles if it means maximizing financial returns (e.g., [29, 30, 31, 32]). In fact, simply priming the concept of money is sometimes sufficient to achieve these effects (e.g., [33, 34, 35, 36, 37]). This line of work suggests that when economic rewards are salient, people are more likely to place weight on their own and others resource needs, such that the moral convictions become less influential on decision making.

Taken together, the evidence indicates both moral convictions and economic rewards are strong motivators for behavior. To date little research has examined how these two motivations may interact in shaping peoples’ attitudes toward resource use. Where the relative influence of both moral and financial considerations has been the focus of previous research, it has investigated how economic gain or moral motives predict attitudes towards energy conservation when both are aligned towards a similar cause [9, 11]. The ways in which these orientations may pull individuals in different directions, such as when financial incentives motivate a focus on personal and others’ resource needs while moral convictions motivate a deontological approach to resource protection, to our knowledge, has not received prior research attention.

### The current study

In the current research we focused on an important social issue–mining. The acceptance of mining activities is a hotly disputed topic around the world [38, 39, 40, 41, 42]. On the one hand, people are concerned about the environmental impact of mining activities: mining activities are inherently disruptive to the environment. For example, mining operations tend to destroy the natural habitat and impact on ground water quality and quantity [43, 44]. On the other hand, many people profit from the mining industry and in some countries mining represents the backbone of the national economy (e.g. Australia). Typically, mining generates employment with corresponding flows of income and wealth accumulation [45, 46, 47].

Mining presents a resource use problem where achieving financial gains often comes with significant environmental costs. This, in turn, provides the foundation for morally motivated opposition. Our aim was to determine whether both moral convictions against mining as well as perceived economic benefits arising from mining may relate to peoples’ acceptance of mining. Given the relatively straightforward relationships between moral convictions, economic benefits, and acceptance of mining, we expected that moral convictions against mining would be associated with a reduced acceptance of mining, while perceived economic benefits from mining would be associated with increased acceptance of mining. Critically, we also predicted that these two orientations would interact, such that moral convictions would be more strongly associated with acceptance of mining when perceived economic benefits were few.

## Method

### Ethics statement

All studies were approved by the CSIRO Social Science Human Research Ethics Committee, within the guidelines of the National Statement on Ethical Conduct in Human Research. Australia survey: Application #023/13, China survey: Application #092/13, Chile survey: Application #061/13. The study procedure was explained to participants and they were informed that by continuing with the questionnaire they were indicating their consent. They were also told that they could withdraw their participation at any time. This consent procedure was approved by the ethics committee.

### Participants and procedure

Professional research survey companies were engaged in each country to recruit participants from both mining and non-mining regions and to conduct the online survey in Australia and China, and door-to-door survey in Chile. Table 1 presents the demographics of the participants. The survey was part of a larger survey investigating citizen’s values, perceptions of mining and relevant stakeholders, and attitudes toward mining. Participants were informed that their responses were anonymous and confidential, and they could withdraw from the survey at any time without penalty.

**Table 1:** Demographics of participants.

Country	Mining region	Non-mining region	Age (years)	Male	Female
	No.	No.	M (SD)	%	%
Australia	650	1940	47.0 (16.63)	46.2%	53.8%
Chile	708	890	47.4 (17.49	47.6%	52.4%
China	992	2328	29.3 (7.93)	48.9%	51.1%
Total	2337	5126	39.2 (16.35)	47.7%	52.3%

### Measures

Moral conviction was measured with 3-items adapted from [48]. Participants were asked to indicate their agreement with each statement on a 7-point scale (*1 = strongly disagree*, *7 = strongly agree*). The items are: “Mining bothers me a lot,” “Mining threatens values that are important to me,” and “My attitude toward mining is a matter of principle.” The internal consistency α ranged from .70 to .87 for the three samples. The items were averaged and a higher score suggests higher moral conviction against mining development.

Economic benefit from mining was measured in two ways. Using a 7-point scale (*1 = strongly disagree*, *7 = strongly agree*), one item asked participants to indicate their agreement with the statement of “The average Australian/Chilean/Chinese is wealthier because of the mining industry.” A second item asked them to indicate their agreement with the statement of “I am better off financially because of the mining boom.” This second item was only used in the surveys for Australia and China. However, we thought it prudent to include this measure as it relates directly to personal economic gain. Thus, we analyzed this separately using a reduced sample (i.e., Australia and China samples only). The two items were, however, significantly correlated (*r* = .44, *p* < .001).

Acceptance of mining was measured with four items on a 5-point scale adapted from [42] in Australia and China surveys. Participants were asked to rate the extent to which they tolerate/accept/approve/embrace mining in Australia/China (*1 = not at all*, *5 = very much so*). The internal consistency coefficients (α) were .89 for Australian sample and .86 for Chinese sample. The four items were averaged and a higher score suggests higher acceptance of mining. In the Chile survey, one item was used to measure participants’ responses to mining on a 5-poing scale (*1 = reject mining*, *2 = tolerate mining*, *3 = accept mining*, *4 = approve mining*, *5 = embrace mining*).

## Results

Preliminary analyses indicated that age was significantly associated with moral conviction (*r* = −.06, *p* < .001) and acceptance of mining (*r* = .14, *p* < .001). That is, older people held lower levels of moral conviction against mining and were more likely to accept mining compared to people with a younger age. In addition, there were gender differences, with males (*M* = 3.36, *SD* = .95) being more likely to accept mining than females (*M* = 3.22, *SD* = .91). As such, age and gender were controlled for in the following analyses.

A series of two-way analysis of covariance (ANCOVA) were conducted to examine the differences for each of the key variables between the three countries by region (i.e., mining and non-mining) while controlling for age and gender.

### Moral conviction

There was a significant interaction between the effects of country and region on moral conviction, *F*(2, 7455) = 12.00, *p* = .001. Simple main effects analysis was conducted to decompose this interaction. For mining regions, participants from both Australia (*M* = 4.07, *SD* = 1.45) and China (*M* = 4.15, *SD* = 1.16) reported significantly higher level of moral conviction against mining than those from Chile (*M* = 3.19, *SD* = 1.55). For non-mining region, participants from China (*M* = 4.13, *SD* = 1.06) reported the highest level of moral conviction, followed by those from Australia (*M* = 3.89, *SD* = 1.38), and participants from Chile (*M* = 2.85, *SD* = 1.58) reported the lowest. In addition, participants from mining regions reported higher levels of moral conviction than those from non-mining regions in Australia and Chile, while participants held the same level of moral conviction in both regions in China.

### Economic benefit from mining

There was a significant interaction between the effects of country and region on economic benefit from mining, *F*(2, 7479) = 48.77, *p* < .001. Simple main effects analysis revealed that for both mining region and non-mining region, participants from Chile (*M* = 5.37, *SD* = 1.60; *M* = 4.42, *SD* = 2.02, respectively) reported the highest level of benefit, followed by those from China (*M* = 4.46, *SD* = 1.51; *M* = 4.27, *SD* = 1.44, respectively), while participants from Australia (*M* = 3.98, *SD* = 1.43; *M* = 4.03, *SD* = 1.45, respectively) reported the lowest level of benefit from mining. In addition, participants from mining regions reported significantly higher level of benefit from mining than those from non-mining regions in both Chile and China, while there was no significant difference between the two regions in Australia.

### Acceptance of mining

The country and region interaction was significant though it did not qualify the main effects, *F*(2, 7494) = 64.05, *p* < .001. Simple main effects analysis suggested that while there were no significant differences in the acceptance level between mining and non-mining regions across all three countries, there were differences between countries. Participants from Chile reported the highest level of acceptance (*M* = 3.51, *SD* = 1.12), followed by those from Australia (*M* = 3.39, *SD* = .88), while participants from China stated the lowest level of acceptance (*M* = 3.09, *SD* = .83).

### The moderating role of economic benefit from mining

In order to examine our focal research question of whether perceived economic benefit from mining moderates the relationship between moral conviction and acceptance of mining, we used hierarchical multiple regression analysis. As there were significant differences among participants from Australia, Chile, and China, variable country was dummy-coded (0, 1, 0 & 0, 0, 1 for each respective country) and controlled for in the regression analysis along with age and gender. Hence, dummy-coded country variables, age, and gender as control variables were entered in the first step; mean-centered moral conviction and economic benefit from mining were entered in the second step; the interaction between moral conviction and benefit from mining was entered in the third step; and acceptance of mining served as the criterion [49]. The hierarchical multiple regression analysis was also conducted for each individual country. The results were consistent across all three countries.

Separate moderation analyses were conducted for mining region only, non-mining region only, and for the complete dataset which includes both mining and non-mining regions. The results were very similar. So the results for the complete dataset were reported here.

**Fig 1:**
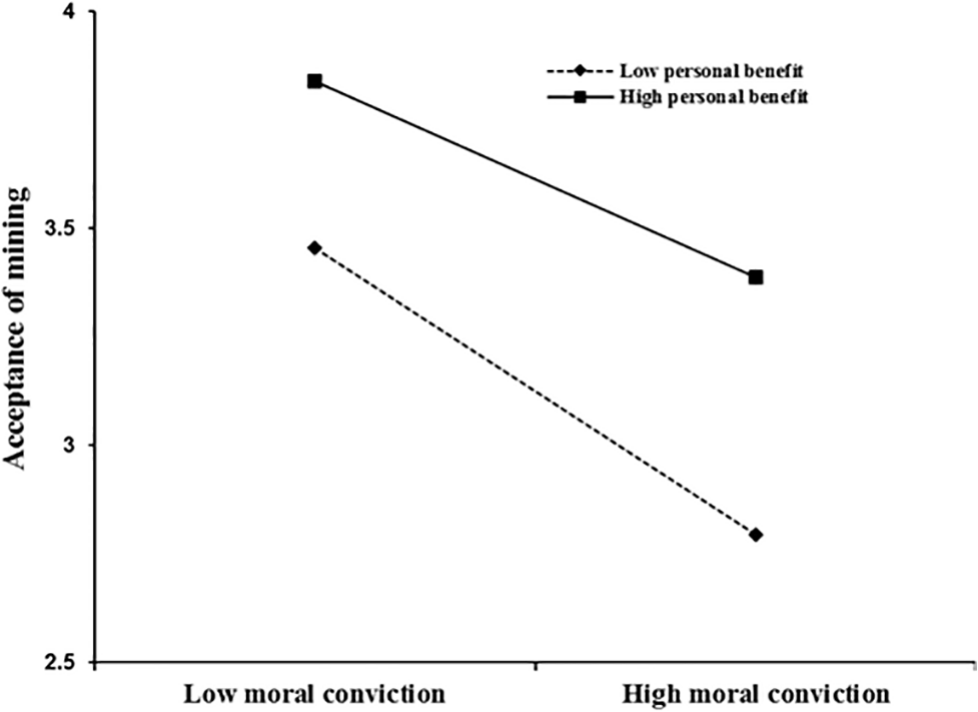
The figure shows the interaction of moral conviction against mining and perceived financial benefit from mining in predicting acceptance of mining. Moral convictions against mining is less strongly associated with acceptance of mining when perceived financial benefits are high.

Moral conviction had a direct effect on acceptance of mining (*β* = −.28, *t*[7435] = - 25.76, *p* < .001), such that higher levels of moral conviction were associated with lower level of acceptance of mining. Perceived economic benefit from mining also had a direct effect on acceptance of mining (*β* = .26, *t*[7435] = 24.33, *p* < .001), such that higher perceived benefit from mining was associated with higher level of mining acceptance. Moreover, in line with our prediction, there was an interaction effect between moral conviction and perceived benefit from mining, *β* = .08, *t*(7435) = 7.20, *p* < .001. Simple slope analyses were conducted to decompose this interaction. As shown in Fig 1, among participants who perceived more economic benefit from mining, moral conviction was negatively associated with acceptance to a lesser degree (*β* = −.19, *t*[7453] = - 12.82, *p* < .001) compared to those who perceived less benefit from mining (*β* = −.30, *t*[7453] = - 20.53, *p* < .001). Two conclusions can be drawn from the findings. First, for those who perceived stronger benefit from mining, they would be more likely to accept mining compared to those who perceived less benefit from mining even when they held the same level of moral convictions against mining. In other words, perceived higher level of benefit enhanced people’s acceptance of mining irrespective of the level of moral convictions they held against mining. Second, when perceived benefit from mining was high, the influence of moral conviction on mining acceptance was weaker.

This same pattern of findings was evident for our measure of personal financial gain on the reduced sample, which applied only to the Australian and Chinese sample (main effect: *β* = .28, *t*[5900] = 24.53, *p* < .001; interaction effect: *β* = .07, *t*[5900] = 5.916, *p* < .001; simple slope high personal benefit: *β* = −.26, *t*[5900] = - 17.21, *p* < .001; simple slope low personal benefit: *β* = −.38, *t*[5900] = - 24.53, *p* < .001)

## Discussion

Both financial incentives and moral convictions are strong motivators of attitudes and decision making. We set out to examine whether economic rewards may shape the influence of moral convictions on attitudes towards resource use. Drawing on three large-scale samples from Australia, China, and Chile, we found that both moral convictions against mining and perceived economic benefit from mining were important predictors of whether people were willing to accept mining development in their own countries. As expected, moral conviction against mining was associated with a reduced level of acceptance of mining while perceived economic benefits were associated with increased acceptance. Importantly, however, we found our predicted interaction between these two factors, such that when perceived economic benefits were high, the relationship between moral conviction and acceptance of mining was weaker. This suggests that although people are motivated to act in accordance with their moral convictions, the salience of economic rewards can motivate them to consider their own and others resources needs, thereby, reducing the influence of their moral convictions in resource decision making. The results of our study are the first to examine how moral convictions may be shaped by the salience of financial rewards in predicting attitudes toward human activities that incur both environmental costs and economic benefits.

Our findings offer important insights into how economic rewards may provide a boundary condition for the effects of moral conviction on resource decision making. Although people may hold personal convictions regarding the exploitation of resource, they at some level are also economic rationalists, influenced by the salience of personal (and other) financial gain. This tension between moral convictions and personal gain was also evident in research showing that such convictions can lead people to act in ways that are not within their immediate economic self-interest [20]. The current findings highlight a different side of this conflict, by showing that salient financial incentives can reduce the influence of moral convictions on decision-making. People are indeed motivated to act in accordance with their moral principles, yet they are also motivated by their own resource needs. Our findings suggest that when resource needs are extreme, personal moral convictions may become relatively inert in motivating human behavior. This possibility is indeed consistent with findings that economic wealth is inversely correlated with environmental protection concerns [25]. When people are concerned about their day-to-day resource needs, they are less inclined to consider larger issues regarding the protection and conservation of natural resource.

Although our study drew on large-scale samples across three different countries, providing confidence in the findings, it also had a number of limitations. The measures for financial gain from mining were based on only single response items due to the need to keep survey items economical. Our focal measure of financial gain for the overall sample was related to the whole country, rather than to people personally. We believe, however, that finding the same pattern of results using a measure that directly tapped personal financial gain with a subset of the overall sample provided some reassurance here. Finally, our data are correlational, therefore making it difficult to infer any causal relationships. Future research might seek to manipulate financial gain or moral conviction in order to examine how each of these motivations might shape the influence of the other in decision making around resource use.

The current research highlights how financial incentives can shape the influence of moral convictions on resource decision-making. The idea that people may be either motivated by personal wealth or moral opposition to environmental exploitation is perhaps not always correct: people may be motivated by both, and when financial incentives are salient, they have the potential to reduce the influence of moral convictions on resource-decision making. The salience of financial rewards may be determined by a number of factors including both chronic (such as poverty) and situational (such as imminent financial gain). Yet, in both cases when the importance of such rewards increases, concerns for the protection and conservation of environmental resources is diminished, even when such concerns are underpinned by moral conviction. Our findings suggest that in order to gain a more complete understanding of how people think about resource use in particular, and social issues more generally, it is important to take account of how short-term gains may shape the ability to consider longer-term consequences.
